# Prediction of Treatment Benefit With Internet-Based Cognitive Behavioral Therapy for Depression

**DOI:** 10.1001/jamanetworkopen.2026.31282

**Published:** 2026-08-28

**Authors:** Cora Schefft, Heiner Stuke, Selin Demir, Maximilian Preiß, Lukas Pezawas, Jakob Kaminski, Björn Meyer, Steffen Moritz, Thomas Berger, Johanne Schröder, Jan Philipp Klein, Stephan Köhler

**Affiliations:** 1 Charité – University Medical Center Berlin, corporate member of Freie Universität Berlin and Humboldt-Universität zu Berlin, Department of Psychiatry and Neurosciences, Berlin, Germany; 2 Centre for Artificial Intelligence in Public Health Research, Robert Koch Institute, Berlin, Germany; 3 Department of Psychiatry and Psychotherapy, Medical University of Vienna, Vienna, Austria; 4 Comprehensive Center for Clinical Neurosciences and Mental Health, Medical University of Vienna, Vienna, Austria; 5 GAIA AG, Hamburg, Germany; 6 Department of Psychiatry and Psychotherapy, University Medical Center Hamburg-Eppendorf, Hamburg, Germany; 7 Department of Clinical Psychology and Psychotherapy, University of Bern, Bern, Switzerland; 8 Institute for Clinical Psychology and Psychotherapy, Department of Psychology, Medical School Hamburg, Hamburg, Germany; 9 Department of Psychiatry, Psychosomatics and Psychotherapy, University of Lübeck, Lübeck, Germany; 10 Center for Brain, Behavior and Metabolism, University of Lübeck, Lübeck, Germany

## Abstract

**Importance:**

Treatment benefit prediction models may help identify which patients will benefit most from internet-based cognitive behavioral therapy (iCBT) for depression; it is unclear whether such models can support useful treatment allocation across programs.

**Objective:**

To develop a model predicting individual treatment benefit from iCBT for depression across programs and to externally validate it in independent samples.

**Design, Setting, and Participants:**

This prognostic study used individual participant data from 5 randomized clinical trials of 3 iCBT programs conducted between 2012 and 2023, with internal validation via repeated nested cross-validation and external validation across 3 independent trials. Participants were adults with depressive symptoms or depressive disorders recruited from community and outpatient clinical settings in Germany and Austria. Analyses were finalized in June 2026.

**Exposure:**

Three iCBT programs (guided or unguided) were compared with treatment as usual with a waitlist or active sham control over 8 to 12 weeks.

**Main Outcomes and Measures:**

The outcome of interest was change in depression severity (assessed using Patient Health Questionnaire–9-item). Elastic net regression, ordinary least squares, and causal forest models were evaluated for agreement between predicted and observed treatment benefit and differences between predicted high- and low-benefit groups.

**Results:**

Of 2037 participants enrolled, 1589 (78.0%; mean [SD] age, 41.5 [11.7] years; 1189 [74.8%] female) with complete outcome data were included. Elastic net regression best predicted outcomes and benefit (*R*^2^ range, 0.19 to 0.50) using 12 predictors. Baseline depression severity was the strongest effect modifier, with each unit increase associated with 0.16 (95% CI, 0.06 to 0.25) points of improvement under treatment. The differences between predicted highest- and lowest-benefit groups excluded zero in only 1 sample (4.37 [95% CI, 0.10 to 8.64] points) and selectively treating patients by model recommendation performed worse than a simple treat-all strategy (range, −2.01 to −0.25 PHQ-9 points).

**Conclusions and Relevance:**

In this prognostic study of treatment benefit from iCBT for depression, more severe depression was associated with greater benefit from iCBT. The model ranked patients by likely benefit but not precisely enough to support model-based targeting. These findings support wider use of iCBT in patients with more severe depression and highlight the need to assess all dimensions of a model’s performance.

## Key Points

**Question:**

Can an individual treatment benefit prediction model for internet-based cognitive behavioral therapy (iCBT) for depression generalize across programs and support individual treatment decisions?

**Findings:**

In this prognostic study of 1589 participants in 5 randomized clinical trials of 3 iCBT programs, an elastic net regression model consistently rank-ordered patients by predicted benefit in external samples, but withholding treatment from patients with low predicted benefit led to worse outcomes at the population level.

**Meaning:**

These findings suggest that benefit stratification by clinical baseline variables was transportable across iCBT programs, but the clinical utility was negative, underscoring the need to evaluate all dimensions of predictive performance in precision psychiatry.

## Teaser

This prognostic study develops and validates a model predicting individual treatment benefit from internet-based cognitive behavioral therapy (CBT) for depression using data from 5 randomized clinical trials of 3 different programs.

## Introduction

Internet-based cognitive behavioral therapy (iCBT) is scalable treatment for depression, with demonstrated efficacy across delivery formats for subclinical and clinical severity ranges.^1,2^ Several health care systems publicly fund iCBT within routine care, and clinical guidelines recommend it for mild to moderate depression.^3,4^ Yet despite this evidence base and available infrastructure, approximately half of patients do not respond to iCBT,^2,5^ dropout rates are substantial, and prescription rates remain low.^6,7^ Identifying which patients benefit most from iCBT using multivariable prediction models could inform more personalized and resource-efficient prescribing.^8,9,10^

Randomized clinical trials (RCTs) are uniquely suited for estimating individual treatment benefit—the individual difference in outcomes between treatment and control, conditional on a patient’s characteristics.^8,11^ Because randomization eliminates dependence between treatment allocation and baseline covariates, these covariates can be used to identify treatment effect modifiers (ie, patient characteristics that enhance or diminish the effect of a treatment).^9,10^ Evaluating such models is methodologically demanding: unlike absolute outcome prediction, true individual treatment benefit is counterfactual, since each participant receives only 1 treatment.^12^ Recent proposals suggest estimates of how well predicted benefits align with observed benefits and whether model-guided allocation improves outcomes at the population level.^13,14^

In the field of precision psychiatry, individual treatment benefit models are rarely evaluated in new data; their clinical utility remaining largely unaddressed.^15^ Model-based treatment rules for iCBT in depression have been proposed for student populations and patients with mild to moderate depression, but neither has been validated externally.^16,17^ To our knowledge, no individual treatment benefit model for iCBT in depression has been developed and externally validated across independent programs and populations.

This study had 3 aims. First, to develop multivariable models on a large, pooled RCT dataset to identify interpretable, prescriptive baseline characteristics predicting differential benefit from iCBT vs control. Second, to test whether the models’ predictions generalize beyond the trials in which they were developed via external validation in independent RCTs spanning similar iCBT programs and across depression severity ranges. Third, across all trials, to assess whether participants stratified by predicted benefit showed corresponding observed benefit (calibration for benefit), and whether differences between strata translated into clinically meaningful decision rules.^13,14^ A model that reliably differentiates high-benefiting from low-benefiting patients could support treatment decisions in iCBT for depression, targeting patients with high predicted benefit while sparing those with low predicted benefit a potentially costly, time-consuming, yet ineffective treatment.

## Methods

This prognostic study was not eligible for additional ethics committee review under the professional code of conduct of the Berlin Chamber of Physicians because data were deidentified prior to receipt. All included trials received ethics approval from their respective review boards, and all participants provided written informed consent. Analyses are reported according to the Transparent Reporting of a Multivariable Prediction Model for Individual Prognosis Or Diagnosis + Artificial Intelligence (TRIPOD+AI) statement.^18^

### Study Design and Data Sources

This prognostic study used an effect modeling approach.^8^ Individual participant data were drawn from 5 RCTs of iCBT for depression conducted in Germany and Austria. Development and 2 validation trials used treatment as usual (TAU) with delayed access to the program as controls (waitlist); 1 trial used an active sham control, creating a different benefit estimand (eMethods 1 in Supplement 1). The development dataset pooled 2 multicenter trials (EVIDENT 1 and 2)^19,20^ evaluating an iCBT tool vs TAU with a waitlist, with recruitment from 2012 to 2015 (1176 participants). A subgroup in EVIDENT 1 received guided treatment. The 3 validation datasets included a single-site trial by Krämer et al^21^ comparing an iCBT tool vs TAU with a waitlist with minimal email support, recruiting from 2019 to 2021 (401 participants), with guided and unguided intervention conditions pooled in this analysis; a single-site trial by Preiss et al^22^ comparing an iCBT tool vs an active sham control, recruiting from 2021 to 2023 (250 participants); and a single-site trial by Moritz et al^23^ comparing an iCBT tool vs TAU with a waitlist, recruiting in 2012 (210 participants).^23^

### Participants

Development trials enrolled adults aged 18 years or older with mild to severe depressive symptoms (assessed using Patient Health Questionnaire–9-item [PHQ-9] score ≥5). Validation trials enrolled adults with major depressive disorder (MDD) or dysthymia (eMethods 1 in Supplement 1).

### Outcomes

The primary outcome was change in depression severity from baseline to 12 weeks (positive values indicate improvement); where 12-week data were unavailable, the closest available assessment (8 or 10 weeks) was used. The development datasets used the PHQ-9 (range 0-27; higher score indicates worse depression).^24^ Published crosswalks converted other depression severity ratings to PHQ-9 equivalents in validation datasets.^25,26^ Only participants with complete outcome data were included (eMethods 2 in Supplement 1).

### Predictors

Baseline variables with more than 35% missingness or high class imbalance (90%:10% ratio) in the development data were removed. Twelve predictors informed by prior literature^2,27^ were included: reported gender, age, relationship status, education, employment, antidepressant use, concurrent psychotherapy, diagnoses of MDD or dysthymia, baseline depression severity, and psychological and physical quality of life. Continuous predictors were centered and scaled prior to model fitting.

### Missing Data

Predictor missingness was low in the development data (0.3%) and was handled by median imputation within each training partition during cross-validation (CV) to prevent data leakage. In external validation datasets, missingness was low overall (2%-4%), but reached 10% to 24% in up to 3 predictors per dataset (eTable 1 in Supplement 1). These were imputed using multiple imputation by chained equations, stacking each validation sample onto the development data, and replacing missing predictor values by values predicted by an imputation model trained on the development sample only (R package mice; 20 imputed datasets, 20 iterations) (eMethods 3 in Supplement 1).^28,29^ Performance was evaluated separately in each imputed dataset and pooled using Rubin rules; sensitivity analyses used median or mode imputation instead.

### Model Development

An elastic net regression (ENR) model was fit on the development data using all 12 predictors and their interactions with treatment assignment. ENR balances L1 and L2 penalties, shrinking coefficients of unimportant predictors toward or to zero, which handles correlated predictors while mitigating overfitting and yielding an interpretable, sparse solution. Because variable selection in ENR is subject to sampling variation, we assessed the stability of selected predictors by refitting the final model in 1000 bootstrap samples of the development dataset, obtaining mean coefficients, 95% CIs, selection frequency, and sign consistency as measures of predictor importance.^30^ Predictors with more than 90% sign consistency and more than 50% selection frequency were considered relevant. Model performance was evaluated both internally in the development sample and externally in the validation samples across 2 dimensions: absolute outcome prediction, and, as the primary outcome, individual treatment benefit prediction.

### Statistical Analysis

#### Evaluation of Absolute Outcome Predictions Internally and Externally

Internally, absolute outcome predictions were evaluated in 10 × repeated 10-fold nested CV (split ratio 9:1), with the mixing (α) and penalty (λ) hyperparameters tuned in the inner CV loop (eFigure 1 in Supplement 1). In each split, the model was refit to the full training partition using the optimal hyperparameters and evaluated in the held-out test partition.

Externally, coefficients from the final model trained on the full development dataset were applied without refitting to obtain outcome predictions in each independent validation sample. Performance was reported as root mean square error (RMSE), *R*^2^, and the intercept and slope of regressing observed on predicted values, with intercept of 0 and slope of 1 representing perfect calibration. ENR was compared with ordinary least squares (OLS) regression.

#### Evaluation of Individual Treatment Benefit Predictions Internally and Externally

Three models were compared for benefit prediction: ENR (α = 1; λ = 0.0713), OLS (α = 0; λ = 0), and causal forests (CF; 2000 trees; √p predictors per split).^31^ For ENR and OLS models, individual treatment benefit was estimated as the predicted outcome under treatment (*t* = 1) minus the predicted outcome under control (*t* = 0) for each individual. CF estimated this benefit directly, without separate outcome predictions for each group. Internally, benefit predictions for the development sample were obtained using 100 × repeated 10-fold CV; externally, the development-trained models were applied without refitting to each independent validation sample.

Performance was assessed using a grouping-by-benefit approach.^13^ Individuals were stratified into quantiles of predicted benefit (11 quantiles in development; 4 in validation), and observed benefit within each quantile (the mean PHQ-9 change difference between treated and untreated individuals) was regressed onto predicted benefit to obtain calibration intercepts, slopes, and *R*^2^. To quantify the magnitude of benefit differentiation, we computed the difference in observed treatment effects between the highest and lowest predicted benefit quantiles. We calculated 95% CIs for intercepts, slopes, and quantile differences by bootstrapping (500 iterations), resampling the data with fixed predictions. We additionally examined whether selectively withholding treatment from the 25% patients predicted to benefit least improved population-level outcomes compared to treating everyone unselectively (decision accuracy analyses) (eMethods 4 in Supplement 1).

All analyses were performed in R version 4.3.1 (R Project for Statistical Computing) using the grf, glmnet, predieval, and caret packages.^13,32,33,34^ All results are reported as point estimates with 95% CIs; a CI excluding zero was used as the criterion for a non-null finding. To test differences in normally distributed baseline values, 2-sided *t* tests were used for continuous variables and χ^2^ tests were used for categorical variables. *P* < .05 was considered statistically significant. Analyses were finalized in June 2026.

## Results

### Sample Characteristics

Among 2037 participants enrolled in 5 RCTs, complete outcome data were available from 1589 participants (78.0%; mean [SD] age, 41.48 [11.66] years; 1189 [74.8%] female). The development dataset included 923 of 1176 (78% retention) participants from EVIDENT 1 (789 participants) and EVIDENT 2 (134 participants) combined. Retention in validation datasets was 364 of 401 participants (90.8%),^21^ 170 of 210 participants (81.0%),^23^ and 132 of 250 participants (52.8%).^22^ Baseline depression severity and diagnostic composition differed across samples (Table 1). Concurrent use of psychotherapy at baseline ranged from 22% to 52%, and use of antidepressant medication ranged from 24% to 71% across samples. iCBT outperformed control conditions in all samples, with mean PHQ-9 differences ranging from 1.37 (95% CI, −0.12 to 2.86) points (*d* = 0.28) in the study by Preiss et al^22^ to 10.37 (95% CI, 8.55 to 12.19) points (*d* = 1.42) in the development dataset.^19,20^

**Table 1.** Baseline Demographic and Clinical Characteristics of Included Participants Across Trials

Variable	Participants, No. (%)			
	EVIDENT 1 and 2^19,20^	Krämer et al^21^	Preiss et al^22^	Moritz et al^23^
Treatment allocation				
Intervention	456 (49.4)	264 (72.5)	57 (43.2)	80 (47.1)
Control	467 (50.6)	100 (27.5)	75 (56.8)	90 (52.9)
Age, mean (SD), y	43.3 (10.9)	37.1 (11.1)	42.6 (11.6)	40.3 (14.0)
Self-reported gender				
Female	650 (70.4)	302 (83.0)^a^	102 (77.3)	135 (79.4)
Male	273 (29.6)	62 (17.0)^a^	30 (22.7)	35 (20.6)
Relationship status				
Partnered	557 (60.3)	133 (36.5)^a^	75 (56.8)	81 (47.6)
Single	366 (39.7)	193 (53.0)^a^	57 (43.2)	89 (52.4)
Missing	NA	38 (10.5)	NA	NA
Education, y				
≤12	259 (28.1)	96 (26.4)	67 (50.8)^a^	94 (55.3)^a^
>12	664 (71.9)	197 (54.1)	65 (49.2)^a^	76 (44.7)^a^
Missing	NA	74 (19.5)	NA	NA
Employment status				
Employed	600 (65.0)	209 (57.4)^a^	102 (77.3)^b^	77 (45.3)^a^
Unemployed	323 (35.0)	66 (18.1)^a^	30 (22.7)^b^	93 (54.7)^a^
Missing	NA	89 (24.0)	NA	NA
Current psychotherapy				
Yes	279 (30.2)	81 (22.3)^b^	48 (36.4)	90 (52.9)^a^
No	644 (69.8)	283 (77.7)^b^	84 (63.6)	39 (22.9)^a^
Missing	NA	NA	NA	41 (24.1)
Current antidepressant				
Yes	361 (39.1)	87 (23.9)^a^	78 (59.1)^a^	120 (70.6)^a^
No	562 (60.9)	277 (76.1)^a^	54 (40.9)^a^	33 (19.4)^a^
Missing	NA	NA	NA	17 (10.0)
Major depressive disorder				
Yes	323 (35.0)	322 (88.5)^a^	132 (100)^a^	140 (82.4)^a^
No	600 (65.0)	42 (11.5)^a^	0^a^	30 (17.6)^a^
Dysthymia				
Yes	358 (38.8)	46 (12.6)^a^	8 (6.1)^a^	139 (81.8)^a^
No	565 (61.2)	318 (87.4)^a^	124 (93.9)^a^	31 (18.2)^a^
QoL psychological subscale, mean (SD)^c^	30.6 (8.0)	49.2 (13.0)^a^	56.5 (16.6)^a^	30.0 (17.5)
QoL physical subscale, mean (SD)^c^	47.0 (9.7)	58.2 (13.5)^a^	45.1 (16.4)	44.2 (16.3)^b^
PHQ-9 baseline, mean (SD)	11.2 (3.5)	18.6 (5.8)^a^	11.7 (4.1)	15.4 (5.5)^a^

Abbreviations: NA, not applicable; PHQ-9, Patient Health Questionnaire-9; QoL, quality of life.

^a^ *P* vs EVIDENT 1 and 2 < .001.

^b^ *P* vs EVIDENT 1 and 2 < .01.

^c^ EVIDENT 1 and 2^19,20^ used the Short Form–12-item, for which scores are norm-referenced against the general population (mean [SD], 50 [10]); values substantially below 50 indicate impaired functioning relative to population norms. Validation datasets used the World Health Organization Quality of Life–Brief, for which domain scores range from 0 to 100 (higher indicates better quality of life). Missingness was 2% and 14.4% for each quality of life domain in EVIDENT 1 and 2^19,20^ sample and Preiss et al^22^ sample, respectively.

### Performance in Absolute Outcome Prediction

ENR showed moderate predictive performance and adequate calibration in the development sample (RMSE, 0.91 SD units; *R*^2^ = 0.18; intercept, −0.07 [95% CI, −1.30 to 1.16]; slope, 1.04 [95% CI, 0.55 to 1.53]) (eTable 3 in Supplement 1) and the validation samples from Moritz et al^23^ (RMSE, 1.00; *R*^2^ = 0.14; intercept, 0.39 [95% CI, −0.88 to 1.66]; slope, 0.77 [95% CI, 0.48 to 1.06]) and Preiss et al^22^ (RMSE, 0.93; *R*^2^ = 0.19; intercept, −1.15 [95% CI, −2.52 to 0.22]; slope, 0.84 [95% CI, 0.55 to 1.13]). In the sample from Krämer et al,^21^ *R*^2^ was higher than in other samples (0.55), indicating a strong linear correlation between predicted and observed outcomes, but calibration was poor: the intercept was below zero (−4.70 [95% CI, −6.23 to −3.17]) and the slope exceeded unity (1.69 [95% CI, 1.53 to 1.85]), indicating that improvements were overestimated for patients with smaller predicted outcome and underestimated for those with larger predicted outcome, likely reflecting this sample’s larger treatment effect and outcome variability relative to the development sample. OLS performed slightly worse; results are reported in eTable 2 in Supplement 1.

Bootstrap analyses indicated greater coefficient stability for ENR than OLS, particularly for interaction terms (Table 2; eTable 3 in Supplement 1). PHQ-9 severity was the dominant treatment effect modifier, selected in 99.6% of bootstrap samples with 100% positive sign consistency: each unit increase in baseline PHQ-9 was associated with 0.16 (95% CI 0.06 to 0.25) points of improvement in the intervention relative to control group. Higher education was associated with lower treatment benefit, but its selection frequency was 63.2%, limiting confidence in its prescriptive value. All remaining predictors were prognostic, predicting symptom reduction regardless of treatment group.

**Table 2.** Elastic Net Regression Coefficients, Variable Selection Frequency, and Sign Consistency Across Bootstrap Samples

Predictor	Coefficient^a^		Selection frequency, %^b^	Positive coefficients, %^c^
	Full sample	Mean (95% CI)		
Main outcomes				
Model intercept	−7.28	−7.25 (−10.51 to −4.17)	100	0
Baseline PHQ-9 severity	0.46	0.47 (0.36 to 0.58)	100	100
Psychological QoL	0.06	0.06 (0.02 to 0.10)	99.6	100
Physical QoL	0.05	0.05 (0.01 to 0.08)	99.8	100
Dysthymia	−0.80	−0.80 (−1.41 to −0.17)	99.3	0
No current psychotherapy	0.51	0.5 (<0.01 to 1.17)	91.9	99.7
Major depressive disorder	−0.33	−0.34 (−1.05 to <0.01)	79.7	1.3
Education >12 y	−0.25	−0.24 (−0.83 to 0.03)	71.1	3.8
Unemployed	−0.22	−0.19 (−0.77 to 0.04)	65.9	5.2
Single relationship status	NS	−0.11 (−0.60 to 0.19)	58.1	16.5
Age	NS	<0.01 (−0.02 to 0.02)	56.0	43.8
No antidepressant medication	NS	0.05 (−0.33 to 0.50)	53.2	68.4
Male sex	NS	0.05 (−0.31 to 0.56)	51.1	66.9
Treatment interaction effects				
Baseline PHQ-9 × intervention	0.16	0.16 (0.06 to 0.25)	99.6	100
Education >12 y × intervention	−0.13	−0.28 (−1.05 to <0.01)	63.2	0.6
Male sex × intervention	0.02	0.11 (−0.29 to 0.74)	48.6	79.6
No antidepressant medication × intervention	0.02	0.12 (−0.17 to 0.74)	46.2	87.9
Single relationship × intervention	NS	0.04 (−0.42 to 0.63)	42.0	61.7
Dysthymia × intervention	NS	0.01 (−0.56 to 0.62)	39.4	51.5
Age × intervention	NS	<0.01 (<0.01 to 0.02)	28.8	99.3
Major depression × intervention	NS	−0.02 (−0.63 to 0.61)	39.0	41.8
No psychotherapy × intervention	NS	−0.04 (−0.64 to 0.37)	32.0	29.7
Psychological QoL × intervention	NS	<0.01 (<0.01 to 0.02)	14.3	76.9
Physical QoL × intervention	NS	<0.01 (<0.01 to <0.01)	5.1	68.6
Intervention group (main effect)	NS	0.01 (<0.01 to <0.01)	2.0	100

Abbreviations: NS, not selected; PHQ-9, Patient Health Questionnaire-9; QoL, quality of life.

^a^ Coefficients are from the full development sample model and bootstrap means across 1000 resamples; 95% CIs reflect the 2.5th and 97.5th percentiles of the bootstrap distribution.

^b^ Selection frequency indicates the proportion of bootstrap samples in which the predictor was retained after elastic net penalization (λ selected by cross-validation).

^c^ Positive coefficients reflects directional consistency across bootstrap samples.

### Performance in Treatment Benefit Prediction

Predicted treatment benefit from ENR ranged from 0 to 4.5 PHQ-9 points across samples (eFigure 2 in Supplement 1). In the development sample, ENR showed limited calibration agreement at 11 quantiles (RMSE, 0.13; *R*^2^  = 0.19; intercept, 1.11 [95% CI, −1.05 to 2.84]; slope, 0.40 [95% CI, −0.66 to 1.61]) (Figure), indicating modest ability to differentiate groups by treatment benefit. OLS and CF models exhibited near-zero or negative slopes in the development sample, suggesting inability to rank groups according to benefit (eFigure 3 and eTable 4 in Supplement 1).

**Figure.**
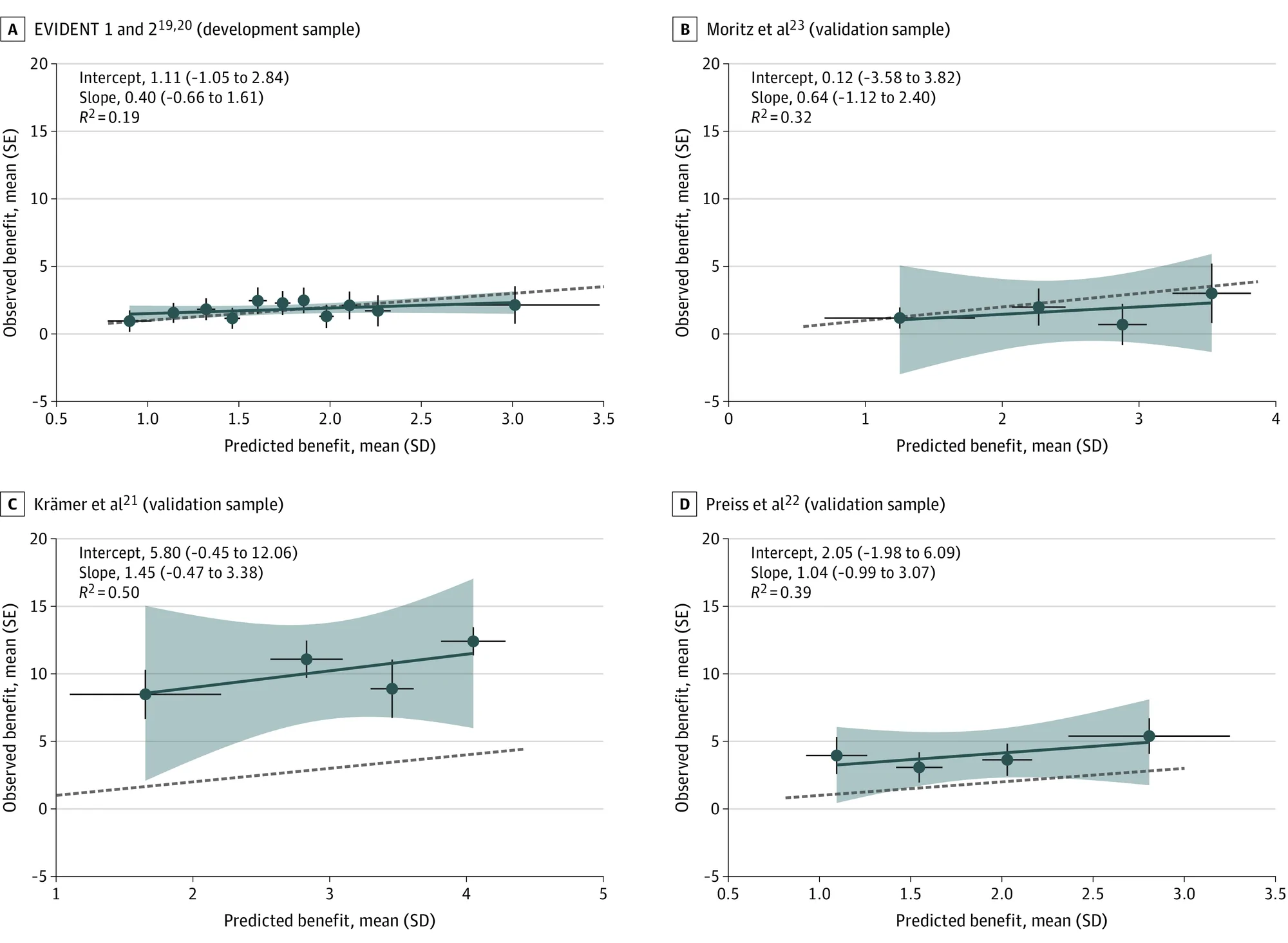
Line Graphs of Calibration for Benefit Plot of the Elastic Net Regression Model Across Samples Each point represents 1 predicted benefit quantile; horizontal error bars show the SD of predicted benefit within the bin; vertical error bars show the SE of the observed treatment effect difference between treatment and control groups. The dashed diagonal indicates perfect calibration (slope, 1; intercept, 0). Fitted lines are derived from linear regression of observed on predicted benefit across bins, with shaded bands showing 95% CIs.

In external validation at 4 quantiles, ENR showed positive calibration slope estimates in all 3 samples (Moritz et al^23^: RMSE, 0.25; *R*^2^ = 0.32; intercept, 0.12 [95% CI, −3.58 to 3.82]; slope, 0.64 [95% CI, −1.12 to 2.40]; Krämer et al^21^: RMSE, 1.37; *R*^2^ = 0.50; intercept, 5.80 [95% CI, −0.45 to 12.06]; slope, 1.45 [95% CI, −0.47 to 3.38]; Preiss et al^22^: RMSE, 0.61; *R*^2^ = 0.39; intercept, 2.05 [95% CI, −1.98 to 6.09]; slope, 1.04 [95% CI, −0.99 to 3.07]). CIs were wide, reflecting the small number of quantile groups, but slope estimates remained stable for other numbers of quantiles, suggesting preserved benefit rank-ordering (eTable 4 in Supplement 1). The large calibration intercepts in Krämer et al^21^ and Preiss et al^22^ reflect systematic underestimation of absolute benefit magnitude in settings with larger average treatment effects than the development sample. OLS performed comparably in the samples from Preiss et al^22^ and Moritz et al^23^; CF produced markedly inflated slopes (range, 2.22 to 3.40). Given its sparsity and more consistent performance, ENR was selected as the primary model.

The difference in observed treatment effects between the predicted highest and lowest benefit quantiles from ENR was 1.34 (95% CI, −1.92 to 4.49) points in the development sample, 1.19 (95% CI −2.51 to 4.89) points in the sample from Preiss et al,^22^ 4.37 (95% CI 0.10 to 8.64) in the sample from Krämer et al,^21^ and 2.34 (95% CI −2.23 to 6.91) in the sample from Moritz et al.^23^ Only Krämer et al^21^ showed a CI excluding zero, suggesting insufficient certainty of stratification in the remaining samples. While patients in the highest quantile benefited by 1.94 to 12.41 points across samples, even patients in the lowest predicted benefit quantile benefited by 1.18 to 8.48 points, suggesting that withholding treatment from this group would likely forgo substantial benefit. This is also reflected in the decision accuracy analyses: withholding from the lowest quartile and selectively treating 75% of the patients predicted to benefit the most leads to worse or equal outcomes compared to unselectively providing treatment to everyone on a population level (range, −2.01 to −0.25 points) (eFigure 4 and eTable 5 in Supplement 1).

## Discussion

In this prognostic study, we developed a model to predict individual treatment benefit from iCBT for depression and evaluated it across multiple dimensions of predictive performance in 3 independent external samples. Despite adequate calibration for absolute outcome prediction, consistently positive calibration for benefit, and evidence of a treatment-specific effect modifier, stratification was insufficiently certain in most samples to support reliable differentiation of patients likely to receive low or high benefit. Only in the largest external sample from the sample by Krämer et al^21^ was a clinically meaningful between-group difference observed. However, even patients in the lowest-benefit quartile improved substantially from iCBT, making treatment withholding clinically unjustifiable. Correspondingly, targeting patients for treatment based on the model’s predictions was mostly detrimental on a population level compared with treating everyone. For future studies, our results suggest that in the presence of a weak effect-modifying signal, high baseline variability, and high average effect size are prerequisites for a model to produce detectable stratification. Trials with restricted inclusion criteria present a structural constraint for precision psychiatry.^35^ Furthermore, our results highlight the importance of evaluating multiple dimensions of a model’s performance, since reliable stratification does not necessarily translate into clinically actionable rules.

Our finding of outcome modification by depression severity replicates a number of previous findings^1,2,36,37^ and carries a positive clinical signal: if benefit increases with severity, iCBT should be considered not only for mild to moderate depression, as currently recommended in guidelines, but also as an adjunct treatment in more severe presentations where face-to-face therapy is unavailable or subject to long waiting times. More broadly, our results support efforts to maximize iCBT availability. The clinician side appears to be a particular lever here: large-scale data from an iCBT website suggest that clinician-prescribed iCBT is used substantially more than self-initiated access.^38^ Engagement could be further increased through artificial intelligence augmentation: a 2026 RCT demonstrated that artificial intelligence–augmented iCBT achieved comparable efficacy with higher engagement and adherence than standard iCBT.^39^ Large language model–based chatbots are also emerging as an efficacious and highly used alternative to iCBT,^40,41^ although their use in populations with mental illness warrants careful regulatory oversight, given the associated risks.^42^

Our finding of ENR’s superiority over OLS or other machine learning methods in this study aligns with prior comparisons favoring regularized regression in moderate-sized clinical datasets.^13,43^ CF’s inflated external calibration slopes, observed here and in a 2023 antidiabetic trial,^43^ suggest overfitting to the development sample’s benefit distribution and limited transportability in smaller external samples.

External validation of benefit prediction models in depression is rare; where reported, results have been mixed. Cross-trial predictions from personalized advantage index models were not significant in either direction,^44^ and most samples were underpowered to detect precision-treatment effect sizes.^45^ Even in a large sample (>8000 students), benefit predictions may not translate into meaningful personalization strategies if effect sizes of the intervention are small to begin with.^46^ In contrast, Furukawa et al^17^ demonstrated personalization gains of 1.41 PHQ-9 points using a prescriptive algorithm within a large trial,^17^ although this finding requires confirmation in an independent sample.

### Limitations

Several limitations should be considered. First, analyses of participants with complete outcome data may introduce selection bias if missingness was related to treatment response. Second, guidance intensity varied across trials and was not randomized at the individual level, precluding its inclusion as a predictor; this unmodeled source of variation may have contributed to miscalibration. Third, all samples were drawn from German-speaking countries, limiting geographical generalizability. Sample race and ethnicity were not systematically recorded across trials, precluding assessment of whether benefit predictions generalize across racial and ethnic groups. Furthermore, interpretation of the sample from the study by Preiss et al^22^ requires caution because substantial attrition and the use of an active sham control may have attenuated observed treatment effects. However, the active sham design is also a strength: since the control condition matched the intervention in structure, engagement, and expectancy, any calibration signal can be attributed to the specific effect of iCBT rather than nonspecific factors.

## Conclusions

In this prognostic study of individual treatment benefit from iCBT for depression, a sparse regularized model differentiated groups of patients by predicted iCBT benefit in a consistent manner across independent trials. This signal was insufficient to support model-guided treatment allocation, underscoring the need to assess all dimensions of predictive performance. Our findings further support offering iCBT adjunctively even in more severe depression and where face-to-face treatment is unavailable or subject to long waiting times.

## Notes

Supplement 1. eMethods 1. Characteristics of Included Samples eMethods 2. Outcomes, Predictors, and Missing Data eMethods 3. Single and Multiple Imputation Procedures eMethods 4. Decision Accuracy Estimators: Definitions and Variance Formulas eFigure 1. Nested Cross-Validation Procedure eFigure 2. Distribution of Benefit Predictions by Model eFigure 3. Calibration for Benefit Plot of Ordinary Least Squares and Causal Forest Models eFigure 4. Population-level Benefit of Model-guided Treatment Allocation versus Universal Treatment across Percentages Withheld eTable 1. Predictor Availability Across Development and Validation Datasets eTable 2. Absolute Outcome Prediction Performance across Internal and External Validation eTable 3. Ordinary Least Squares Regression Coefficients and Bootstrap Stability for the Outcome Prediction Model eTable 4. Calibration of Treatment Benefit Prediction Models in External Validation Samples eTable 5. Population-Level Benefit of Withholding Treatment from the Lowest-Predicted-Benefit Subgroup, Across Withheld Proportions

Supplement 2. Data Sharing Statement
