# Supplementary material for: Prediction of Treatment Benefit With Internet-Based Cognitive Behavioral Therapy for Depression
Source: JAMA Netw Open. 2026 Aug 28;9(8):e2631282. doi: 10.1001/jamanetworkopen.2026.31282 (PMC13525460; doi:10.1001/jamanetworkopen.2026.31282)
Supplement: Supplementary file 1 [file jamanetwopen-e2631282-s001.pdf]

Supplemental Online Content

Schefft C, Stuke H, Demir S, et al. Prediction of treatment benefit with internet-based cognitive behavioral therapy for depression. *JAMA Netw Open*. 2026;9(8):e2631282. doi:10.1001/jamanetworkopen.2026.31282

eMethods 1. Characteristics of Included Samples
eMethods 2. Outcomes, Predictors, and Missing Data
eMethods 3. Single and Multiple Imputation Procedures
eMethods 4. Decision Accuracy Estimators: Definitions and Variance Formulas
eFigure 1. Nested Cross-Validation Procedure
eFigure 2. Distribution of Benefit Predictions by Model
eFigure 3. Calibration for Benefit Plot of Ordinary Least Squares and Causal Forest Models
eFigure 4. Population-level Benefit of Model-guided Treatment Allocation versus Universal Treatment across Percentages Withheld
eTable 1. Predictor Availability Across Development and Validation Datasets
eTable 2. Absolute Outcome Prediction Performance across Internal and External Validation
eTable 3. Ordinary Least Squares Regression Coefficients and Bootstrap Stability for the Outcome Prediction Model
eTable 4. Calibration of Treatment Benefit Prediction Models in External Validation Samples
eTable 5. Population-Level Benefit of Withholding Treatment from the Lowest-Predicted-Benefit Subgroup, Across Withheld Proportions

This supplemental material has been provided by the authors to give readers additional information about their work.

© 2026 Schefft C et al. *JAMA Network Open*. This is an open access article distributed under the terms of the CC-BY-NC-ND License, which does not permit alteration or commercial use, including those for text and data mining, AI training, and similar technologies.

eMethods 1. Characteristics of Included Samples

Study Characteristics

Characteristic	EVIDENT [1]	Deprexis Severe [2]	Selfapy [3]	Deprexis Validation Sample [4]	edupression.com[5] (Preprint)
Country	Germany (5 sites)	Germany	Germany	Germany	Austria (Vienna)
N Randomized	1013 (509 intervention + 504 treatment as usual)	163 (78 intervention + 85 treatment as usual/waitlist)	401 (151 guided intervention + 150 unguided intervention + 100 wait list with supportive emails)	210 (105 intervention + 105 wait list)	250 (125 intervention + 125 active sham control)

Inclusion/Exclusion Criteria

Criteria	EVIDENT Trial 1	EVIDENT Trial 2	Selfapy	Deprexis Validation Sample	edupression.com (Preprint)
Severity	PHQ-9: 5-14 (mild-moderate)	PHQ-9: ≥15 (severe)	BDI-II ≥10 (mild-severe)	Self-reported depression	ICD-10: F32.0/1, F33.0/1 (mild-moderate), PHQ≥5
Age	18+	18+	18-65	18-65	18-65
Exclusion	Suicidality, bipolar, schizophrenia	Suicidality, bipolar, schizophrenia	Suicidality, bipolar, schizophrenia	Bipolar, psychosis, suicidality	Bipolar, psychosis, suicidality, substance use disorder, treatment resistance
Concurrent Treatment	Allowed	Allowed	Allowed	Allowed	Allowed

Intervention Characteristics

Study	Program	Type	Guidance	Control Description[1]
**EVIDENT Trial 1**	Deprexis	10 CBT modules	Minimal (weekly email support for PHQ 10-14)	Treatment as usual (TAU) - continued usual care (psychotherapy, medication, or none) Between baseline and psychotherapy 32 – 35 % received psychotherapy, 27% outpatient psychiatric treatment, 1-2 % inpatient psychiatric treatment, 50% Antidepressant medication. Offered access after last follow-up.

© 2026 Scheft C et al. *JAMA Network Open*. This is an open access article distributed under the terms of the CC-BY-NC-ND License, which does not permit alteration or commercial use, including those for text and data mining, AI training, and similar technologies.

**EVIDENT Trial 2**	Deprexis	10 CBT modules	Unguided	TAU; around 50% on antidepressants at baseline, around 50% in psychotherapy
**Selfapy**	Selfapy	6 core + 6 optional modules	One Guided arm (weekly phone conversations, discussing content of the app, meeting therapist once)	TAU/Waitlist with weekly standardized mindfulness exercises via email. email for 24 weeks, designed to control for expectancy effects. Participants free to access other treatments. Notably: 24% of control participants started psychotherapy during the trial versus only 3–4% in intervention groups.
**Deprexis Validation Sample**	Deprexis	10 CBT modules	Completely unguided	TAU /Passive waitlist
**Edupression (Preprint)**	edupression.com	150+ topics, 27 exercises, MBC	Completely unguided	Active sham control - structurally matched app with identical interface but replaced all therapeutic content with non-specific wellness material (lifestyle tips, motivational content, relaxing music) and provided no feedback on symptom inputs. It thereby controlled for expectancy, engagement, and attention without delivering any active treatment. Both conditions additional to TAU: around 53% antidepressant use and 38% psychotherapy.

[1] Treatment as usual for depression in central European German-speaking countries usually involves weekly psychotherapy sessions and/or monthly to quarterly visits to primary care doctors or psychiatrists for antidepressant treatment regimens covered by public insurances. More rarely, patients are hospitalized in inpatient or partial hospitalisation programs.

eMethods 2. Outcomes, Predictors, and Missing Data

We included all predictors that were available for more than 65% of the participants with low class imbalance (<90/10) in both development and validation datasets. In the Deprexis validation dataset two predictors were missing and were replaced by proxy variables: Dysthymia was replaced by a specifier that marked the depressive episode as “chronic” (≥2 year duration) and Relationship was approximated by the baseline score of item 20 of the WHOQOL-BREF[6] scale (“How satisfied are you with your personal relationships?”, values ≥ 3 were classified as partnered, < 3 as single). Quality of life (QoL) was assessed using two different instruments in the development and the validation datasets. Although QoL subscales were among the most stable predictors of absolute outcome, QoL-by-treatment interaction terms were not selected during ENR development, suggesting that instrument heterogeneity had limited influence on benefit predictions specifically.

Before model fitting, continuous predictors were centred and scaled as automated in *glmnet* and then back transformed to original scale.[7]

In 63 cases (17.3%) of the Selfapy sample, QIDS-SR data was missing at post-intervention. We therefore replaced the missing values by converting available HRSD-24 sum scores to QIDS-SR sum scores following published conversion tables.[8]

The distribution of missing data is shown in eTable 1.

© 2026 Schefft C et al. *JAMA Network Open*. This is an open access article distributed under the terms of the CC-BY-NC-ND License, which does not permit alteration or commercial use, including those for text and data mining, AI training, and similar technologies.

eMethods 3. Single And Multiple Imputation Procedures

We compared two strategies of handling missing predictors:

1) Multiple imputation with chained equations using mice[9]: Missing predictor data in validation datasets were handled by using multiple imputation by chained equations (MICE) in a stacked approach in which each validation sample was appended to the development data prior to imputation. We imputed 20 datasets with a maximum of 20 iterations for each external validation set. Logistic regression was specified for binary variables, predictive mean matching for continuous variables. Absolute outcome prediction, calibration for benefit and population-level benefit were assessed in each imputed dataset. Performance metrics RMSE, R^2, MAE were obtained by averaging over imputed datasets; intercepts and slopes were obtained by pooling according to Rubin's rules. Validation data were excluded from fitting the imputation models using the *ignore* argument in the *mice* package, ensuring that imputation models were estimated on development data only.[10] The outcome variable was excluded from all imputation models consistent with the deployment scenario in which outcome data are unavailable at prediction time.[11]
2) Single median and mode imputation based on parameters obtained in the development sample. The rationale behind this imputation strategy was to apply the single imputation approach used in the development sample to all external validation samples. At deployment, only distribution parameters of the development sample are known and therefore the only basis for imputation.

eMethods 4. Decision Accuracy Estimators: Definitions And Variance Formulas

Decision accuracy was evaluated using population-level benefit measures following Efthimiou et al.[12]

The measure $PB1_{IPW}$ quantifies the benefit of treating only model-recommended participants versus treating everyone. Treating everyone was chosen as the most informative comparator since the original trials showed positive effect sizes, suggesting treatment is effective on average. When this is the case, the question of interest is: how much can we gain at the population level by treating model-recommended patients instead of treating everyone, as suggested by the positive average treatment effect?

To assess population-level benefit we imagined a limited access scenario, where participants were ranked by predicted benefit and the bottom proportion (p) was withheld,

© 2026 Schefft C et al. *JAMA Network Open*. This is an open access article distributed under the terms of the CC-BY-NC-ND License, which does not permit alteration or commercial use, including those for text and data mining, AI training, and similar technologies.

with $PB1_{IPW}$ evaluated across withheld proportions from 5% to 50% (in steps of 5%); primary results are reported at p = 10%, 20%, 25% and 75% in eTable 5. This ensures that exactly p% of participants are withheld regardless of the absolute scale of predicted benefit, allowing comparison across samples with different average treatment effects.

The scenario answers the question whether the model can identify the subgroup least likely to benefit when resources are constrained. Results are reported as point estimates with 95% CI.

To obtain $PB1_{IPW}$, all patients are classified into four groups based on their predicted benefit ($\hat{B}$) and the treatment received. A threshold (T) separates patients with predicted benefit above the threshold from those below. Patients in G1 and G4 received the treatment recommended by the model (optimal); patients in G2 and G3 did not (suboptimal). Group sizes n1 + n2 correspond to participants with predicted benefit above threshold ($\hat{B} > T$), and n3 + n4 to participants at or below threshold ($\hat{B} \leq T$).

	$\hat{B} > T$	$\hat{B} \leq T$
**t = 1**	G1 (optimal)	G3 (suboptimal)
**t = 0**	G2 (suboptimal)	G4 (optimal)

Notation

Symbol	Meaning
$\hat{B}i$	Predicted treatment benefit for patient i (positive = favours treatment)
n_k	Number of patients in group Gk
π	Propensity $\frac{(n_1 + n_3)}{(n_1 + n_2 + n_3 + n_4)}$

Estimator $PB1_{IPW}$

$PB1_{IPW}$ compares the mean outcome under model-recommended treatment to the mean outcome under active treatment for everyone (t = 1 counterfactual). To adjust for randomization imbalances, all means are inverse propensity weighted (IPW).

Point estimate:

© 2026 Schefft C et al. *JAMA Network Open*. This is an open access article distributed under the terms of the CC-BY-NC-ND License, which does not permit alteration or commercial use, including those for text and data mining, AI training, and similar technologies.

$$\mathrm{PB1}_{IPW} = \frac{\frac{\sum_{G1} y_i}{\pi} + \frac{\sum_{G4} y_i}{1-\pi}}{\frac{n_1}{\pi} + \frac{n_4}{1-\pi}} - \frac{\sum_{G1} y_i + \sum_{G3} y_i}{n_1 + n_3}$$

Variance:

$$Var(PB1_{IPW}) = n_1 Var(y_{G1}) w_1^2 + n_4 Var(y_{G4}) w_4^2 + n_3 Var(y_{G3}) w_3^2$$

$$D = \frac{n_1}{\pi} + \frac{n_4}{1-\pi}$$

$$w_1 = \frac{1}{\pi D} - \frac{1}{n_1 + n_3}$$

$$w_4 = \frac{1}{(1-\pi)D}$$

$$w_3 = \frac{1}{n_1 + n_3}$$

eFigure 1. Nested Cross-Validation Procedure

Schematic of the 10-times repeated 10-fold nested cross-validation used in the development dataset (Deprexis, n = 923). In each outer fold, 90% of participants were used for training (inner loop) and 10% for testing. The inner loop performed 10-fold cross-validation to tune hyperparameters (α, λ) for elastic net regression. Optimal hyperparameters were selected as medians across 100 iterations (α = 1.0, λ = 0.0713). Performance metrics reported for internal validation represent averages across all 100 outer test partitions. p = Number of predictors.

© 2026 Schefft C et al. *JAMA Network Open*. This is an open access article distributed under the terms of the CC-BY-NC-ND License, which does not permit alteration or commercial use, including those for text and data mining, AI training, and similar technologies.

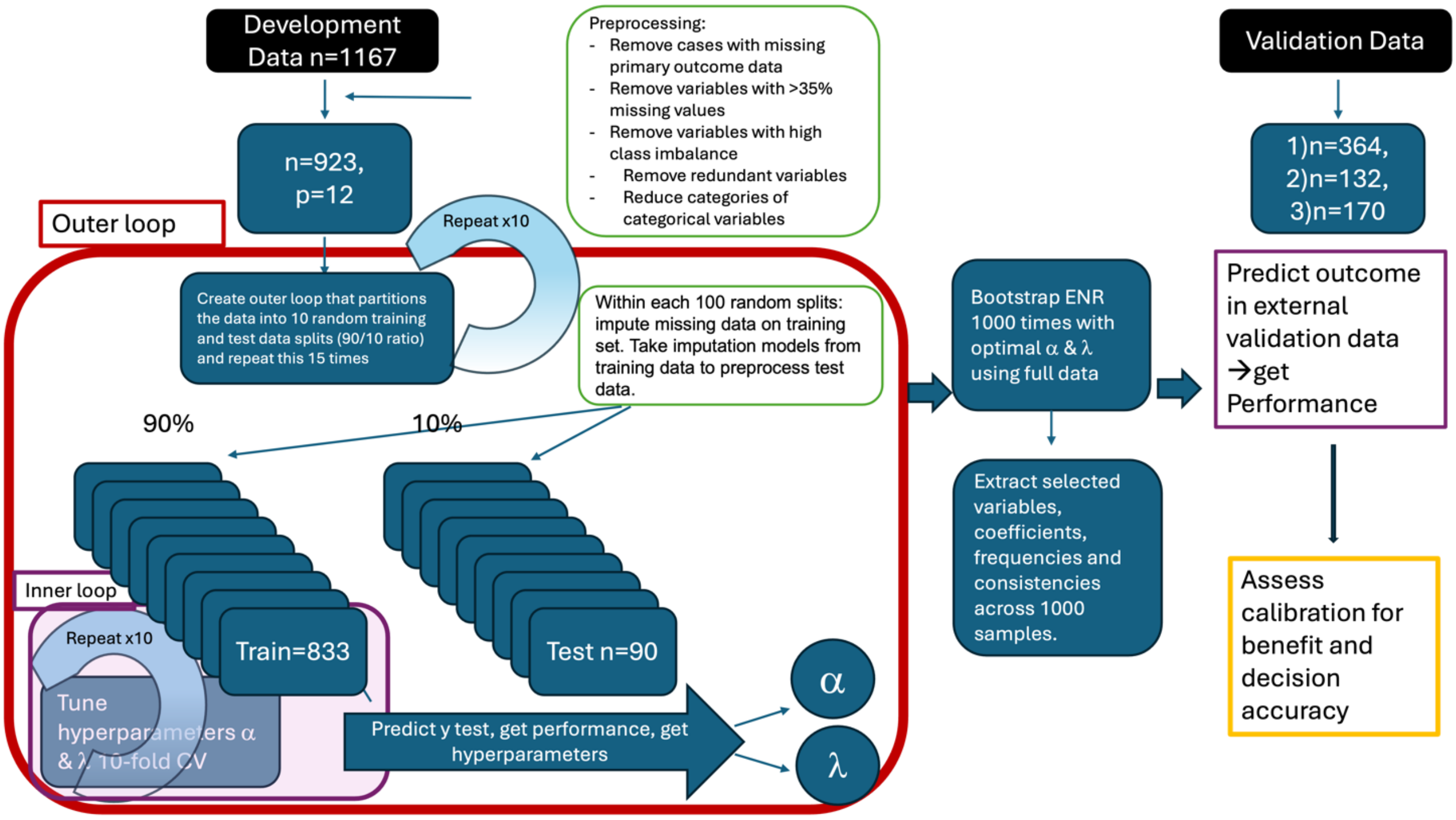

© 2026 Schefft C et al. *JAMA Network Open*. This is an open access article distributed under the terms of the CC-BY-NC-ND License, which does not permit alteration or commercial use, including those for text and data mining, AI training, and similar technologies.

eFigure 2. Distribution Of Individual Treatment Benefit Predictions across Development and Validation Samples.

Density plots of ENR-predicted individual treatment benefit (PHQ-9 change score units, treatment minus control) for each sample. Predictions were derived from the final model trained on the full development dataset (Deprexis, shaded) and applied without retraining to three external validation samples. Vertical dashed lines indicate a small effect size (d ≥ 0.25; 1.14 PHQ-9 points) threshold anchored to the development sample pooled within-group SD (4.57). ENR = elastic net regression; PHQ-9 = Patient Health Questionnaire-9.

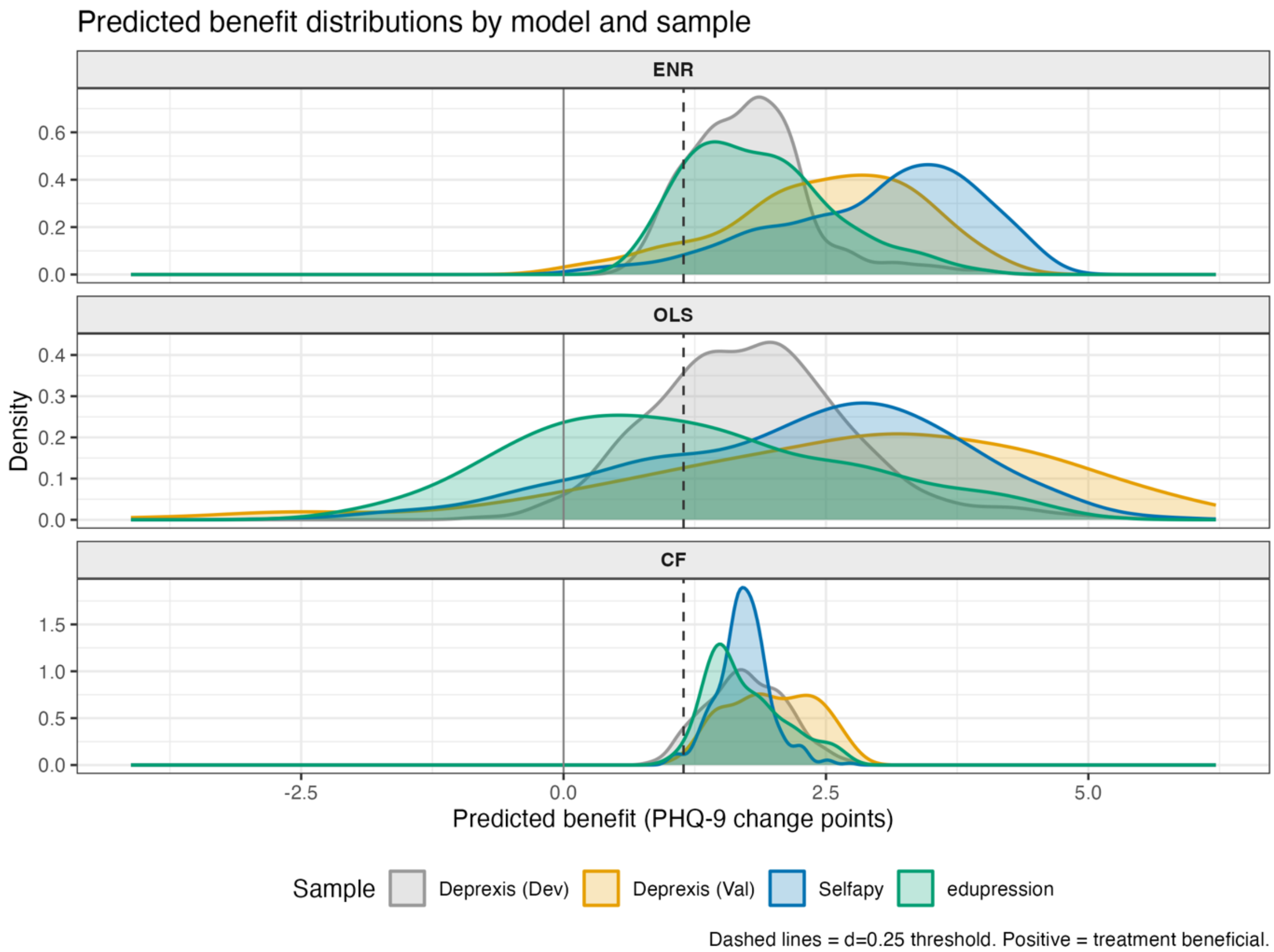

© 2026 Schefft C et al. *JAMA Network Open*. This is an open access article distributed under the terms of the CC-BY-NC-ND License, which does not permit alteration or commercial use, including those for text and data mining, AI training, and similar technologies.

eFigure 3. Calibration for Benefit Plot of Ordinary Least Squares and Causal Forest Models

Calibration for benefit of the OLS (top row) and causal forest (bottom row) models in the development sample (Deprexis Dev, shaded, k=11 bins) and three external validation samples (Deprexis Val, Selfapy, edupression, k=4 bins each). Each point represents one predicted benefit quantile; horizontal error bars show the standard deviation of predicted benefit within the bin; vertical error bars show the standard error of the observed treatment effect difference between treatment and control arms. The dashed diagonal indicates perfect calibration (slope=1, intercept=0). Fitted lines are derived from linear regression of observed on predicted benefit across bins, with shaded bands showing 95% confidence intervals. Calibration intercept, slope (both with 95% bootstrap CI), and R^2 are shown per panel. External validation estimates are MICE-pooled across 20 imputations with Rubin's rules applied to standard errors.

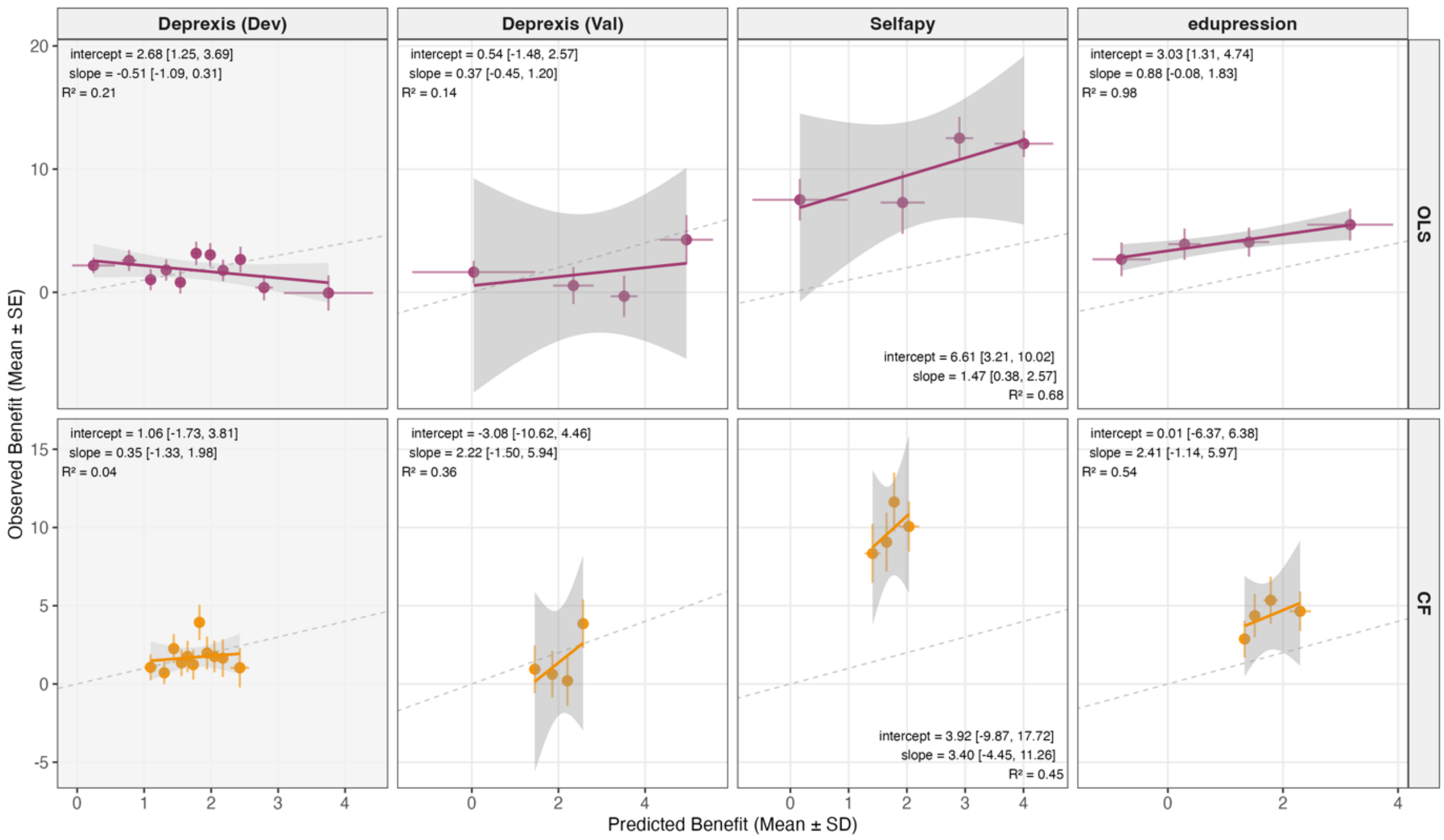

© 2026 Schefft C et al. *JAMA Network Open*. This is an open access article distributed under the terms of the CC-BY-NC-ND License, which does not permit alteration or commercial use, including those for text and data mining, AI training, and similar technologies.

eFigure 4. Population-level Benefit of Model-guided Treatment Allocation versus Universal Treatment across Percentages Withheld.

Each panel shows $PB1_{IPW}$ (incremental population benefit versus universal treatment, in PHQ-9 points) as a function of the proportion of participants withheld from treatment — specifically, those with the lowest predicted benefit. Positive values indicate that model-guided allocation outperforms a treat-all strategy; negative values indicate harm relative to universal treatment. The teal dashed vertical line marks 25% withheld as a reference threshold, vertical dashed line marks zero. Shaded bands represent 95% confidence intervals. For the Deprexis development sample, benefit predictions derive from 10-fold cross-validation averaged across 100 repeats; for external validation samples (Selfapy, edupression, Deprexis [Val]), PB1 estimates and confidence intervals were pooled across 20 multiple imputations using Rubin's rules. Sample sizes: Deprexis (Dev) n = 923; Selfapy n = 364; edupression n = 132; Deprexis (Val) n = 170.

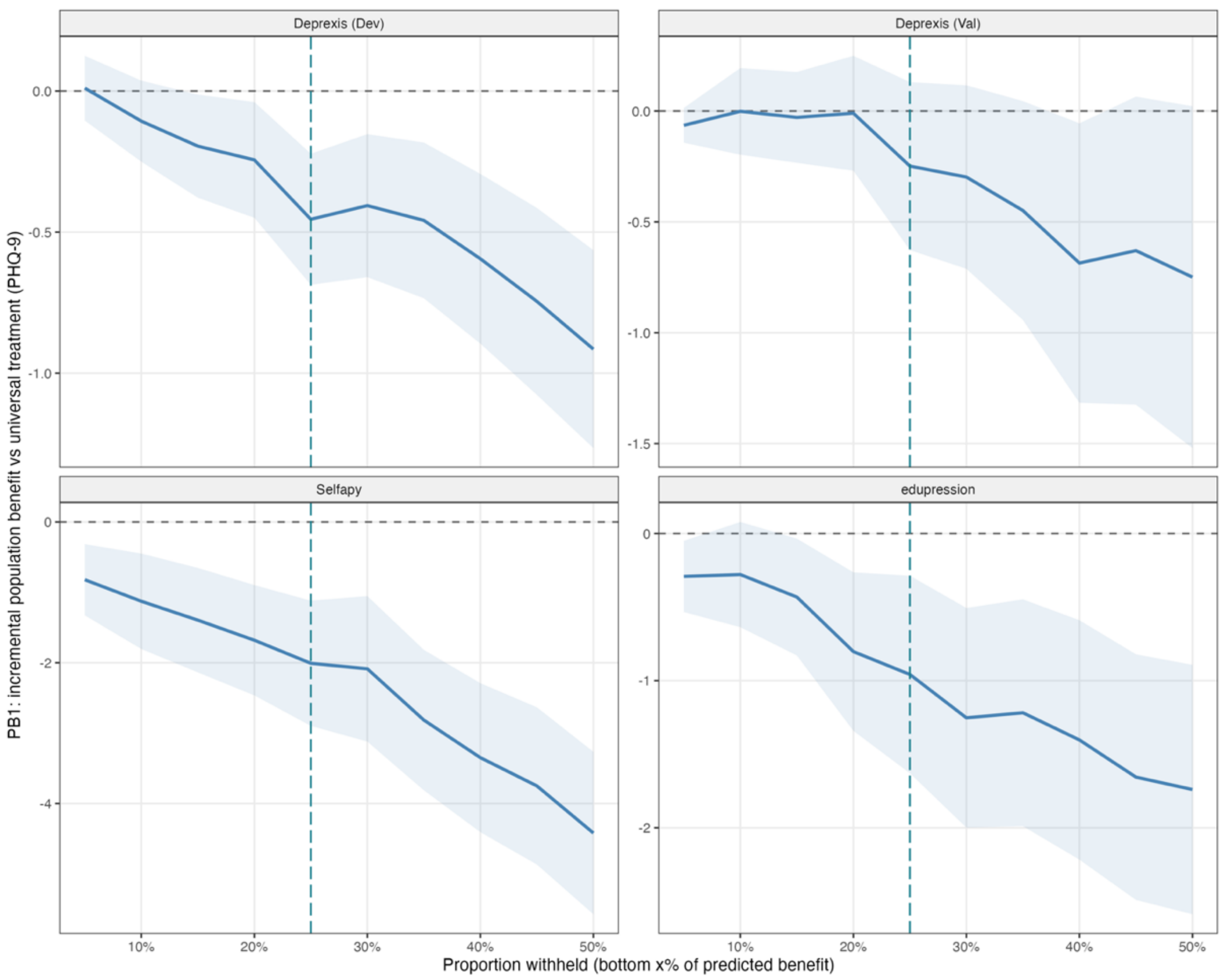

© 2026 Schefft C et al. *JAMA Network Open*. This is an open access article distributed under the terms of the CC-BY-NC-ND License, which does not permit alteration or commercial use, including those for text and data mining, AI training, and similar technologies.

eTable 1. Predictor Availability across Development and Validation Datasets

Predictor	Development Deprexis (n=923)	Selfapy (n=364)	edupression (n=132)	Deprexis validation (n=170)
Baseline depression severity	0 (0%)	0 (0%)	0 (0%)	0 (0%)
Age	0 (0%)	0 (0%)	0 (0%)	0 (0%)
Sex	0 (0%)	0 (0%)	0 (0%)	0 (0%)
School education	0 (0%)	74 (19.5%)	0 (0%)	0 (0%)
Relationship status	0 (0%)	38 (10.0%)	0 (0%)	0 (0%)
Employment status	0 (0%)	89 (24.0%)	0 (0%)	0 (0%)
Current antidepressant	0 (0%)	0 (0%)	0 (0%)	17 (10.0%)
Current psychotherapy	0 (0%)	0 (0%)	0 (0%)	41 (24.0%)
MDD diagnosis	0 (0%)	0 (0%)	0 (0%)	0 (0%)
Dysthymia diagnosis	0 (0%)	0 (0%)	0 (0%)	0 (0%)
QoL physical	18 (2.0%)	0 (0%)	19 (14.4%)	0 (0%)
QoL psychological	18 (2.0%)	0 (0%)	19 (14.4%)	0 (0%)
Sum of total missing values	36/10153 (0.3%)	201/4368 (4.6%)	38/1584 (2.4%)	58/2040 (2.84%)

Values indicate number (percentage) of participants with missing data. MDD = major depressive disorder; QoL = quality of life.

© 2026 Schefft C et al. *JAMA Network Open*. This is an open access article distributed under the terms of the CC-BY-NC-ND License, which does not permit alteration or commercial use, including those for text and data mining, AI training, and similar technologies.

eTables 2. Absolute Outcome Prediction Performance across Internal and External Validation

Model performance metrics for predicting PHQ-9 change scores from baseline to 8-12 weeks post-randomization. RMSE = root mean squared error; MAE = mean absolute error; R^2 = coefficient of determination; CI = confidence interval; SD = standard deviation. *Standardized metrics calculated using the development sample SD (4.65) for all samples to allow direct comparison. A) Multiple imputation results, RMSE, MAE, R^2 represent means (SD) across 20 imputed datasets, Intercepts and Slopes represent means with 95% CI based on pooling according to Rubin's rules. B) For the development sample (Deprexis Dev), only single imputation results are reported. Development sample metrics represent mean (SD) across 100 cross-validation test partitions; external validation metrics are single estimates. Predictor missingness was low (≤2% per predictor); missing values were handled by median imputation applied separately within each training fold during nested cross-validation. Multiple imputation and complete case analyses were therefore not performed for this sample.

A) Stacked multiply imputed data

Sample	Model	RMSE (mean, SD)	RMSE (SD units)* (mean, SD)	MAE (mean, SD)	R^2 (mean, SD)	Calibration Intercept [SE]	Calibration Slope [SE]	SD of PHQ-9 change
Selfapy	ENR	6.52 (0.01)	1.40 (0.00)	5.49 (0.01)	0.55 (0.00)	-4.7 (0.78)	1.69 (0.08)	8.65
Selfapy	OLS	6.53 (0.01)	1.40 (0.00)	5.37 (0.01)	0.48 (0.00)	-3.73 (0.84)	1.45 (0.08)	8.65
edupression	ENR	4.36 (0.02)	0.93 (0.00)	3.36 (0.02)	0.19 (0.01)	-1.15 (0.70)	0.84 (0.15)	4.43
edupression	OLS	4.83 (0.02)	1.03 (0.00)	3.69 (0.02)	0.13 (0.01)	-0.46 (0.71)	0.59 (0.14)	4.43
Deprexis (Val)	ENR	4.60 (0.00)	1.00 (0.00)	3.59 (0.00)	0.14 (0.00)	0.39 (0.65)	0.77 (0.15)	4.92
Deprexis (Val)	OLS	4.69 (0.01)	1.01 (0.00)	3.65 (0.01)	0.13 (0.00)	0.9 (0.59)	0.64 (0.13)	4.92

B) Single median and mode imputation parameter derived from the development sample

Sample	Model	RMSE	RMSE (SD units)*	MAE	R^2	Calibration Intercept [SE]	Calibration Slope [SE]	SD of PHQ-9 change
Deprexis (Dev)	ENR	4.22 (0.27)	0.91 (0.06)	3.26 (0.21)	0.18 (0.08)	-0.07 (0.63)	1.04 (0.25)	4.65
Deprexis (Dev)	OLS	4.25 (0.28)	0.91 (0.06)	3.28 (0.21)	0.17 (0.07)	0.15 (0.58)	0.95 (0.23)	4.65
Selfapy	ENR	6.52	1.40	5.49	0.55	-4.70 (0.78)	1.69 (0.08)	8.65
Selfapy	OLS	6.53	1.41	5.38	0.48	-3.71 (0.84)	1.45 (0.08)	8.65
edupression	ENR	4.39	0.94	3.37	0.19	-1.10 (0.69)	0.82 (0.15)	4.43
edupression	OLS	4.87	1.05	3.70	0.12	-0.42 (0.72)	0.58 (0.14)	4.43
Deprexis (Val)	ENR	4.60	0.99	3.60	0.14	0.32 (0.66)	0.77 (0.15)	4.92

© 2026 Schefft C et al. *JAMA Network Open*. This is an open access article distributed under the terms of the CC-BY-NC-ND License, which does not permit alteration or commercial use, including those for text and data mining, AI training, and similar technologies.

Deprexis (Val)	OLS	4.72	1.02	3.73	0.14	0.58 (0.62)	0.65 (0.13)	4.92

eTable 3. Ordinary Least Squares Regression Coefficients and Bootstrap Stability for the Outcome Prediction Model

Coefficients are bootstrap means across 1000 resamples of the development sample. 95% CIs reflect the 2.5th and 97.5th percentiles of the bootstrap distribution. Negative (%) and Positive (%) indicate the directional consistency of the coefficient across bootstrap samples. Interaction terms denote predictor × treatment group allocation. PHQ-9 = Patient Health Questionnaire-9; CI = confidence interval.

Predictor	Mean Coefficient (1000 Bootstraps)	95% CI	Negative (%)	Positive (%)
**Main Effects**				
Model intercept	−10.02	−14.45 to −5.46	100	0
Intervention group (main effect)	0.51	0.39 to 0.65	100	100
Male sex	0.09	0.03 to 0.15	100	99.9
Age	0.08	0.03 to 0.12	100	99.9
Education >12 years	−0.92	−1.71 to −0.09	100	1.3
Unemployed	0.89	−0.03 to 1.85	100	96.9
No current psychotherapy	−0.35	−1.35 to 0.56	100	25.1
No antidepressant medication	−0.33	−1.23 to 0.55	100	23.0
Major depressive disorder	−0.13	−1.02 to 0.66	100	38.9
Dysthymia	−0.19	−0.98 to 0.56	100	33.7
Psychological quality of life	−0.01	−0.05 to 0.03	100	36.0
Physical quality of life	−0.11	−0.95 to 0.72	100	39.2
Baseline PHQ-9 severity	0.04	−0.79 to 0.92	100	51.8
**Treatment Interaction Effects**				
Intervention × Male sex	0.20	0.01 to 0.40	100	98.0
Intervention × Age	−0.52	−1.82 to 0.76	100	19.9
Intervention × Education >12 years	0.27	−0.93 to 1.39	100	66.9
Intervention × Unemployed	0.42	−0.72 to 1.66	100	75.0
Intervention × No current psychotherapy	0.10	−1.00 to 1.22	100	56.0
Intervention × No antidepressant medication	0.02	−1.11 to 1.14	100	50.2
Intervention × Major depressive disorder	0.01	−0.04 to 0.07	100	70.2
Intervention × Dysthymia	−0.32	−1.59 to 1.10	100	29.6
Intervention × Psychological quality of life	−0.54	−1.82 to 0.73	100	19.6
Intervention × Physical quality of life	−0.03	−0.11 to 0.05	100	22.9
Intervention × Baseline PHQ-9 severity	−0.03	−0.10 to 0.04	100	20.1

© 2026 Schefft C et al. *JAMA Network Open*. This is an open access article distributed under the terms of the CC-BY-NC-ND License, which does not permit alteration or commercial use, including those for text and data mining, AI training, and similar technologies.

eTable 4. Calibration of Treatment Benefit Prediction Models in External Validation Samples

Intercepts and slopes from calibration regression of observed group-mean treatment effect on mean predicted benefit within quantile bins (k = number of bins). RMSE = root mean squared error of calibration (standardised by pooled SD). For Deprexis (Dev), predictions derive from 10-fold cross-validation (100 repeats); For external validation samples, MI uses stacked multiple imputation (m = 20) with Rubin's rules; median/mode imputed results were nearly identical and are therefore not shown. ENR = elastic net regression; OLS = ordinary least squares; CF = causal forest; SE = Standard Error.

© 2026 Schefft C et al. *JAMA Network Open*. This is an open access article distributed under the terms of the CC-BY-NC-ND License, which does not permit alteration or commercial use, including those for text and data mining, AI training, and similar technologies.

Sample	Method	N groups	Intercept (95% CI)	Slope (95% CI)	RMSE	R2
**Deprexis (Dev)**	ENR	4	1.57 (-0.61 to 3.36)	0.13 (-1.01 to 1.38)	0.11	0.21
		18	0.56 (-1.05 to 2.53)	0.71 (-0.46 to 1.64)	0.24	0.14
		50	0.75 (-1.40 to 3.08)	0.65 (-0.79 to 1.93)	0.48	0.03
	OLS	4	2.32 (1.04 to 3.62)	-0.33 (-1.02 to 0.42)	0.29	0.27
		11	2.68 (1.25 to 3.69)	-0.51 (-1.09 to 0.31)	0.39	0.21
		18	2.17 (0.98 to 3.45)	-0.23 (-1.01 to 0.39)	0.42	0.02
		50	2.03 (0.88 to 3.26)	-0.17 (-0.91 to 0.40)	0.54	0.01
	CF	4	1.14 (-1.80 to 4.39)	0.32 (-1.61 to 2.05)	0.16	0.02
		11	1.06 (-1.73 to 3.81)	0.35 (-1.33 to 1.98)	0.14	0.04
		18	1.34 (-1.54 to 4.32)	0.17 (-1.63 to 1.84)	0.22	0
		50	1.58 (-1.94 to 4.46)	-0.03 (-1.68 to 1.93)	0.45	0
**edupression**	ENR	3	1.58 (-2.62 to 5.77)	1.28 (-0.83 to 3.38)	0.59	0.52
		5	2.58 (-1.41 to 6.58)	0.67 (-1.36 to 2.71)	0.65	0.09
		10	2.59 (-1.55 to 6.73)	0.65 (-1.46 to 2.75)	0.71	0.05
	OLS	3	3.15 (1.42 to 4.88)	0.84 (-0.13 to 1.80)	0.78	0.97
		4	3.03 (1.31 to 4.74)	0.88 (-0.08 to 1.83)	0.75	0.98
		5	3.31 (1.58 to 5.05)	0.59 (-0.37 to 1.55)	0.76	0.85
		10	3.48 (1.67 to 5.28)	0.47 (-0.51 to 1.44)	0.94	0.12
	CF	3	0.08 (-6.92 to 7.08)	2.40 (-1.50 to 6.30)	0.7	0.53
		4	0.01 (-6.37 to 6.38)	2.41 (-1.14 to 5.97)	0.69	0.54
		5	0.97 (-5.37 to 7.32)	1.89 (-1.62 to 5.39)	0.69	0.44
		10	1.47 (-4.93 to 7.87)	1.69 (-1.82 to 5.20)	0.88	0.1
**Selfapy**	ENR	3	4.96 (-1.06 to 10.97)	1.69 (-0.11 to 3.50)	1.28	1
		5	6.17 (0.55 to 11.80)	1.30 (-0.42 to 3.01)	1.36	0.87
		10	6.80 (0.40 to 13.21)	1.07 (-0.89 to 3.03)	1.46	0.18
	OLS	3	8.02 (4.52 to 11.52)	1.00 (-0.22 to 2.21)	1.32	0.8
		4	6.61 (3.21 to 10.02)	1.47 (0.38 to 2.57)	1.3	0.68
		5	7.26 (4.02 to 10.51)	1.15 (0.07 to 2.24)	1.3	0.47
		10	6.75 (3.76 to 9.74)	1.33 (0.35 to 2.31)	1.4	0.29
	CF	3	2.77 (-12.09 to 17.64)	4.13 (-4.35 to 12.61)	1.19	0.8
		4	3.92 (-9.87 to 17.72)	3.40 (-4.45 to 11.26)	1.19	0.45
		5	3.25 (-10.48 to 16.98)	3.84 (-3.98 to 11.67)	1.2	0.55
		10	3.86 (-9.19 to 16.90)	3.42 (-3.99 to 10.83)	1.23	0.14
**Deprexis (Val)**	CF	3	-3.48 (-11.34 to 4.39)	2.42 (-1.47 to 6.32)	0.19	0.85
		5	-3.08 (-10.27 to 4.11)	2.21 (-1.31 to 5.74)	0.31	0.37
		10	-1.34 (-8.40 to 5.71)	1.31 (-2.23 to 4.84)	0.42	0.1
	ENR	3	0.54 (-3.31 to 4.39)	0.44 (-1.35 to 2.23)	0.21	0.89
		4	0.12 (-3.58 to 3.82)	0.64 (-1.12 to 2.40)	0.25	0.32
		5	-0.11 (-3.64 to 3.42)	0.75 (-0.97 to 2.46)	0.39	0.15
		10	-0.25 (-4.03 to 3.53)	0.80 (-1.03 to 2.64)	0.47	0.12
	OLS	3	1.23 (-0.94 to 3.39)	0.17 (-0.71 to 1.05)	0.4	0.21
		4	0.54 (-1.48 to 2.57)	0.37 (-0.45 to 1.20)	0.5	0.14
		5	0.70 (-1.23 to 2.62)	0.36 (-0.43 to 1.14)	0.53	0.12

© 2026 Schefft C et al. *JAMA Network Open*. This is an open access article distributed under the terms of the CC-BY-NC-ND License, which does not permit alteration or commercial use, including those for text and data mining, AI training, and similar technologies.

Sample	Method	N groups	Intercept (95% CI)	Slope (95% CI)	RMSE	R2
		10	0.60 (-1.33 to 2.52)	0.41 (-0.41 to 1.24)	0.57	0.13

eTable 5. Population-Level Benefit of Withholding Treatment From the Lowest-Predicted-Benefit Subgroup, Across Withheld Proportions

This table reports $PB1_{IPW}$ — the incremental population-level benefit of withholding treatment from the proportion (p) of participants with the lowest predicted treatment benefit, relative to treating everyone — at four withheld proportions (10%, 20%, 25%, 75%) in each sample. Negative values indicate that model-guided allocation performed worse than universal treatment; positive values would indicate model-guided allocation outperformed treating everyone. An asterisk denotes a 95% CI excluding zero. $PB1_{IPW}$ was negative and statistically distinguishable from zero across all samples at most withheld proportions, with the exception of Deprexis (Val), where intervals included zero throughout.

Sample	p_withhold	n	n_withheld	sig	PB1 (95% CI)
Deprexis (Dev)	0.1	923	93		-0.11 [-0.25, 0.04]
Deprexis (Dev)	0.2	923	185	*	-0.24 [-0.45, -0.04]
Deprexis (Dev)	0.25	923	231	*	-0.45 [-0.69, -0.22]
Deprexis (Dev)	0.75	923	692	*	-1.25 [-1.70, -0.80]
Selfapy	0.1	364	35	*	-1.13 [-1.80, -0.45]
Selfapy	0.2	364	73	*	-1.68 [-2.46, -0.90]
Selfapy	0.25	364	91	*	-2.01 [-2.90, -1.12]
Selfapy	0.75	364	273	*	-7.51 [-9.15, -5.87]
edupression	0.1	132	13		-0.28 [-0.64, 0.08]
edupression	0.2	132	26	*	-0.80 [-1.34, -0.26]
edupression	0.25	132	32	*	-0.96 [-1.63, -0.29]
edupression	0.75	132	98	*	-2.62 [-3.68, -1.56]
Deprexis (Val)	0.1	170	17		-0.00 [-0.20, 0.19]
Deprexis (Val)	0.2	170	34		-0.01 [-0.27, 0.25]
Deprexis (Val)	0.25	170	42		-0.25 [-0.63, 0.13]
Deprexis (Val)	0.75	170	126		-0.79 [-1.89, 0.31]

© 2026 Schefft C et al. *JAMA Network Open*. This is an open access article distributed under the terms of the CC-BY-NC-ND License, which does not permit alteration or commercial use, including those for text and data mining, AI training, and similar technologies.

References

1. Klein JP, Berger T, Schröder J, et al. Effects of a psychological internet intervention in the treatment of mild to moderate depressive symptoms: results of the EVIDENT study, a randomized clinical trial. *Psychother Psychosom.*2016;85(4):218-228. doi:10.1159/000445355
2. Meyer B, Bierbrodt J, Schröder J, et al. Effects of an internet intervention (Deprexis) on severe depression symptoms: a randomized clinical trial. *Internet Interv.* 2015;2(1):48-59. doi:10.1016/j.invent.2014.12.003
3. Kramer R, Köhne-Volland L, Schumacher A, Köhler S. Efficacy of a web-based intervention for depressive disorders: a randomized clinical trial comparing guided and unguided self-help with waitlist control. *JMIR Form Res.* 2022;6(4):e34330. doi:10.2196/34330
4. Moritz S, Schilling L, Hauschildt M, Schröder J, Treszl A. A randomized clinical trial of internet-based therapy in depression. *Behav Res Ther.* 2012;50(7-8):513-521. doi:10.1016/j.brat.2012.04.006
5. Preiss M, Stamenkovic K, Schimmerl B, et al. Efficacy and safety of a digital therapeutic for depression: a randomized clinical trial with active sham control. *SSRN.* Published 2025. doi:10.2139/ssrn.5213394
6. WHOQOL Group. Development of the World Health Organization WHOQOL-BREF quality of life assessment. *Psychol Med.* 1998;28(3):551-558. doi:10.1017/S0033291798006667
7. Friedman JH, Hastie T, Tibshirani R. Regularization paths for generalized linear models via coordinate descent. *J Stat Softw.* 2010;33(1):1-22. doi:10.18637/jss.v033.i01
8. Rush AJ, Trivedi MH, Ibrahim HM, et al. The 16-item Quick Inventory of Depressive Symptomatology (QIDS), clinician rating (QIDS-C), and self-report (QIDS-SR): a psychometric evaluation in patients with chronic major depression. *Biol Psychiatry.* 2003;54(5):573-583. doi:10.1016/s0006-3223(02)01866-8
9. van Buuren S, Groothuis-Oudshoorn K. mice: multivariate imputation by chained equations in R. *J Stat Softw.*2011;45(3):1-67. doi:10.18637/jss.v045.i03
10. Hoogland J, van Barreveld M, Debray TPA, et al. Handling missing predictor values when validating and applying a prediction model to new patients. *Stat Med.* 2020;39(25):3591-3607. doi:10.1002/sim.8682
11. Sisk R, Sperrin M, Peek N, van Smeden M, Martin GP. Imputation and missing indicators for handling missing data in the development and deployment of clinical prediction models: a simulation study. *Stat Methods Med Res.*2023;32(8):1461-1477. doi:10.1177/09622802231165001
12. Efthimiou O, Hoogland J, Debray TPA, et al. Measuring the performance of prediction models to personalize treatment choice. *Stat Med.* 2023;42(8):1188-1206. doi:10.1002/sim.9665

© 2026 Schefft C et al. *JAMA Network Open*. This is an open access article distributed under the terms of the CC-BY-NC-ND License, which does not permit alteration or commercial use, including those for text and data mining, AI training, and similar technologies.
