# Supplementary material for: Prediction of Treatment Benefit With Internet-Based Cognitive Behavioral Therapy for Depression
Source: JAMA Netw Open. 2026 Aug 28;9(8):e2631282. doi: 10.1001/jamanetworkopen.2026.31282 (PMC13525460; doi:10.1001/jamanetworkopen.2026.31282)
Supplement: Supplementary file 2 [file jamanetwopen-e2631282-s002.pdf]

Data Sharing Statement

Schefft. Prediction of Treatment Benefit With Internet-Based Cognitive Behavioral Therapy for Depression. *JAMA Netw Open*. Published August 28, 2026.
doi:10.1001/jamanetworkopen.2026.31282

Data
Data available: No

Additional Information
Explanation for why data not available: The datasets generated and analyzed during the current study are not publicly available due to data protection regulations and the terms of the individual study agreements, but deidentified participant-level data may be made available to qualified researchers upon reasonable request to the corresponding author, subject to institutional data transfer agreements and ethical approval. Analysis code is available at github.com/coraschefft.
